# Fungal Keratitis Caused by Aureobasidium pullulans: A Case Report

**DOI:** 10.7759/cureus.108733

**Published:** 2026-05-12

**Authors:** Suwarna Suman, Arushi Kumar

**Affiliations:** 1 Department of Ophthalmology, All India Institute of Medical Sciences, Jodhpur, Jodhpur, IND; 2 Department of Otorhinolaryngology, Sanjay Gandhi Postgraduate Institute of Medical Sciences, Lucknow, IND

**Keywords:** aureobasidium pullulans, dematiaceous fungi, fungal keratitis, opportunistic fungal infection, phaeohyphomycosis

## Abstract

*Aureobasidium pullulans* is a dematiaceous fungus and a rare causative agent of fungal keratitis. We report a case of *Aureobasidium pullulans* keratitis that did not respond to conventional therapy despite two months of treatment for a corneal ulcer. A 52-year-old male patient presented with complaints of pain and redness of the right eye (OD) for two months. There was no history of any ocular trauma or surgery, or any other significant ocular and systemic illness. In the left eye (OS), a laser peripheral iridotomy was done 15 days ago. The patient was under treatment for a OD fungal corneal ulcer without any significant improvement after two months. Fungal culture isolated *Aureobasidium* *pullulans* as the causative infectious organism. The patient was treated successfully with topical natamycin and systemic itraconazole.

## Introduction

Fungal keratitis, or keratomycosis, has a worldwide distribution but is most common in tropical and subtropical regions [1,2]. After *Fusarium* and *Aspergillus *species, the dematiaceous fungi are the third most common group of etiologic agents [1]. The dematiaceous fungi are a heterogeneous group of filamentous fungi having brown melanin or melanin-like pigments in the cell walls of their hyphae, conidia, or both [1,3]. Curvularia species are the most commonly recovered dematiaceous moulds from patients with mycotic keratitis in the United States [1]. *Aureobasidium pullulans* is a rare causative agent of fungal keratitis among dematiaceous fungi. In a case series of 30 patients with fungal keratitis from Australia, a single case of *Aureobasidium pullulans* keratitis (3%) and 12 cases of *Curvularia *species (39%) were reported [2]. In a study from Nepal, four (10.4%) cases of *Aureobasidium pullulans* keratitis among 38 cases of fungal keratitis (25 cases of isolated fungal growth and 13 cases mixed with other bacteria and fungi) were isolated from culture [4]. Dematiaceous keratitis commonly presents as a low-grade, dry-looking, raised corneal ulcer with minimal inflammation [1, 5]. A pigmented slough is usually seen [5]. Infection usually follows the ocular trauma, or there may be a history of ocular surgery or contact lens use. It is common in diabetic and immunosuppressed patients [1,5]. Symptoms include ocular redness, watering, photophobia, and decreased vision, which are common in fungal keratitis [1]. The clinical diagnosis of dematiaceous keratitis is difficult, as clinical manifestations of fungal keratitis are similar regardless of the causative agent [1, 5]. Culture is generally considered the gold standard for identifying the causative agent to confirm the diagnosis and enables microbial susceptibility testing for appropriate treatment, but results may take weeks. A delay in diagnosis increases the duration of treatment and also affects the outcome. Further, there is limited available data on *Aureobasidium *keratitis, especially regarding its diagnosis and management. Only a few case reports following ocular trauma or surgery are published in the literature [6]. 

We report a case of *Aureobasidium pullulans* keratitis in a 52-year-old male patient who did not respond to conventional therapy for a corneal ulcer for two months. There was no history of any ocular trauma or surgery, or any other significant ocular and systemic illness. This case report describes the patient’s clinical course, with emphasis on early diagnosis and management by using a systematic approach to achieve an optimal outcome.

## Case presentation

A 52-year-old male office worker presented with complaints of pain, redness, watering, and diminution of vision of the right eye (OD) for two months. There was no history of any ocular trauma or surgery. However, he reported frequent visits to the countryside to look after his farms and domestic pets. In the left eye (OS), a laser peripheral iridotomy was done 15 days ago. However, the indication for the procedure was not mentioned in the previous prescription. There was no history of diabetes mellitus, hypertension, or any other significant ocular and systemic illness. Laboratory investigations, including microbiological tests, were not performed. The patient was undergoing treatment for a fungal corneal ulcer in the OD with natamycin (pimaricin) 5% eye drops, moxifloxacin-tobramycin eye drops administered every two hours, amphotericin B eye drops six times daily, and itraconazole eye ointment twice daily. In addition, brinzolamide and brimonidine eye drops were administered three times daily in both eyes. He was prescribed acetazolamide 250 mg twice daily, diclofenac with serratiopeptidase twice daily, and pantoprazole 40 mg once daily in the morning. However, the patient did not notice a significant improvement and consulted at this institute.

**Figure 1:**
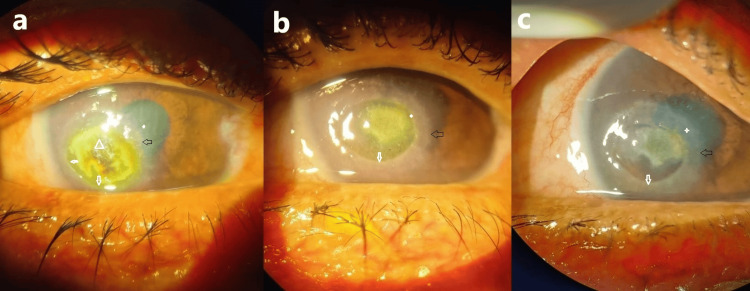
Clinical photograph showing (a) a corneal ulcer with greyish exudates (white triangle), surrounded by stromal infiltration and oedema (black arrow) at presentation, (b) a decrease in necrotic cellular debris after one week, and (c) a transparent floor and smoother edges (white arrow) after two weeks in the regression stage. The centre of the pupil is marked (white diamond).

At presentation, his unaided visual acuity was OD 3/60 and OS 6/12. With correction, it improved to 6/9 in the OS and showed no improvement in the OD. In the OD, there was mild conjunctival congestion and an ulcer of about 4.5 x 4.0 mm in size, surrounded by about one mm of stromal infiltration and oedema. The floor of the ulcer was covered with exudate with a greyish hue. There were Descemet’s folds in the surrounding cornea and pigments on the back of the cornea. The pupil was normal in size and sluggishly reacting to light. Intraocular pressure (IOP) was OD 16 mmHg and OS 17 mmHg by the non-contact tonometer (Figure 1a). In the OS, a peripheral iridotomy was present superotemporally. The rest of the ocular examination was within normal limits.

Based on the clinical findings (ulcer floor covered with greyish exudates, slow progression, and minimal inflammation), a provisional clinical diagnosis of dematiaceous fungal keratitis was made. Other more common causes of fungal keratitis were excluded: *Fusarium *keratitis is more severe, progresses deeply, and would have been perforated within two months; *Aspergillus *keratitis is moderately severe but generally responsive to treatment. To identify the causative organism, microbiological investigations were recommended.

Routine blood investigations, including blood sugar, liver, and renal function tests, were unremarkable. Corneal scraping was performed for smears, and Gram and Giemsa staining, potassium hydroxide (KOH) wet preparations, and bacterial and fungal cultures were recommended. Homatropine 2% eye drops were administered three times daily, and timolol-dorzolamide eye drops were administered twice daily in addition to the previous topical antibiotic and antifungal medications. Brinzolamide and brimonidine eye drops and the oral medications were discontinued. The patient was advised to revisit after three days with reports. No aerobic bacteria were seen after 48 hours of incubation. The KOH mount showed moderately thin, septate, hyaline fungal hyphae with acute-angle branching. Tablet itraconazole 100 mg twice a day was added along with topical medications (eye drop natamycin hourly and eye drop atropine thrice a day), and a weekly review was advised. After one week, signs of regression of the corneal ulcer were noted: a line of demarcation around the ulcer, formed by leucocytes, and a decrease in necrotic cellular debris. There was an initial enlargement of the ulcer due to a decrease in necrotic material. The floor and edges become smoother and transparent after two weeks (Figures 1b-1c).

**Figure 2:**
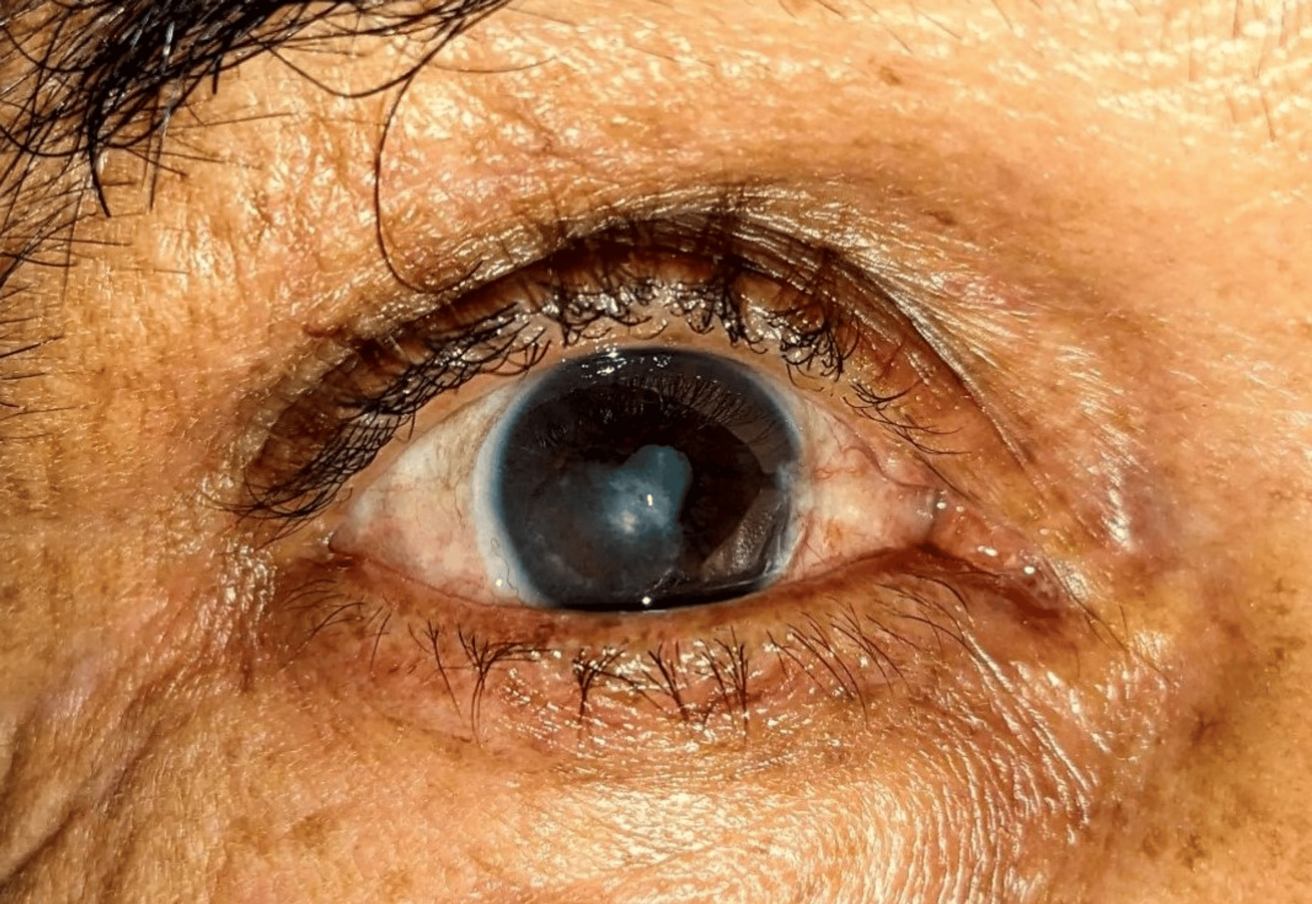
Healed corneal ulcer.

After three weeks of treatment, signs of healing of the corneal ulcer were noted: the ulcer had reduced in size to approximately 2.6 x 2.2 mm, and the surrounding infiltration and swelling had disappeared. Visual acuity was 5/60 OD and 6/18 OS. However, in the OS, an increase in IOP (36 mmHg) with a +2 cell anterior chamber reaction was noticed. Sabouraud dextrose agar revealed growth of A*ureobasidium pullulan*s after three weeks of incubation. In the OD, the same treatment was continued. For the OS, acetazolamide 250 mg tablets were prescribed three times daily, along with moxifloxacin-dexamethasone eye drops hourly, atropine eye drops three times daily, and timolol-dorzolamide eye drops twice daily. A weekly review was recommended, and medications were tapered according to response. After six weeks of treatment, the fluorescein stain was negative; visual acuity was OD 5/60 and OS 6/12, and IOP was OD 18 mmHg and OS 25 mmHg. Oral itraconazole was discontinued, and the topical antifungal eye drop was tapered in four weeks. After 10 weeks of treatment, the corneal ulcer healed with minimal scarring; visual acuity was OD 6/24 and OS 6/12, and IOP was OD 18 mmHg and OS 19 mmHg (Figure 2). The patient is under investigation and treatment for anterior uveitis with secondary glaucoma in the OS.

The clinical course and treatment over the past 10 weeks are summarised in Table 1.

**Table 1:** Summary of clinical course and treatment timeline. BCVA: best-corrected visual acuity; IOP: intraocular pressure; OD: right eye; OS: left eye; CU: corneal ulcer; ED: eye drops; TID: three times daily; BID: twice daily; KOH: potassium hydroxide; CCC: circumcorneal congestion; OU: both eyes; QID: four times daily

Time	BCVA	IOP (mmHg)	Clinical Features	Investigations	Treatment
At presentation	OD: 3/60, OS: 6/9	OD: 16, OS: 17	OD: CU 4.5 x 4.0 mm in size, stromal infiltration, greyish exudates, Descemet’s membrane folds with few pigments on the endothelium. OS: peripheral iridotomy	Routine blood Investigations, corneal scraping for smears and culture	OD: ED homatropine 2% TID, ED timolol + dorzolamide BID, ED natamycin 5% two hourly, ED tobramycin +moxifloxacin two hourly, ED amphotericin B six times/day, eye ointment itraconazole BID
Three days later	OD 3/60, OS 6/12	OD:19, OS: 21	OD: same, OS: same	KOH mount: thin, septate, hyaline fungal hyphae with acute-angle branching. Bacterial culture: No aerobic bacterial growth after 48 hours of incubation	OD: ED natamycin hourly, ED atropine sulfate 1% TID, ED timolol + dorzolamide BID. Tablet Itraconazole 100 mg BID
One week later	OD 3/60, OS 6/12	OD:24, OS:21	OD: a line of demarcation around the ulcer, a decrease in necrotic cellular debris, and enlargement of the ulcer	Bacterial culture: No aerobic bacteria were seen after 48 hours of incubation	OD: same treatment (patient missed ED timolol + dorzolamide)
Two weeks later	OD 3/60, OS 6/12	OD: 18, OS: 19	OD decrease in necrotic material, floor, and base, transparent and smooth	Fungal culture: no growth	OD: same treatment
Three weeks later	OD 5/60, OS 6/18	OD: 19, OS: 36	OD: CU 2.6 x 2.2 mm in size. OS: CCC, +2 cells anterior chamber reaction	Fungal culture: Sabouraud dextrose agar revealed growth of *Aureobasidium pullulans *after three weeks of incubation	OD: ED atropine BID, rest, the same treatment. OS: ED moxifloxacin + dexamethasone hourly, ED atropine TDS, ED timolol + dorzolamide BID. Tablet acetazolamide 250 mg TID
Four weeks later	OD: 5/60, OS: 6/18	OD: 19, OS: 22	OD: CU 1.8 x 1.6 mm in size. OS: CCC, +1 cells anterior chamber reaction		OD: ED atropine once daily, rest, the same treatment. OS: ED moxifloxacin + dexamethasone 2 hourly, ED atropine BID, ED timolol + dorzolamide BID.
Six weeks later	OD: 5/60, OS: 6/12	OD: 18, OS: 25	Healed corneal ulcer (fluorescein stain negative) OS: occasional cells in the anterior chamber		OD: ED natamycin tapered 6; 4;3; two times/day in four weeks. OS: ED moxifloxacin + dexamethasone six times/day, ED timolol + dorzolamide BID. Tablet acetazolamide 250 mg TID. OU: ED atropine discontinued. Tablet itraconazole discontinued
10 weeks later	OD: 6/24, OS: 6/12	OD: 18, OS: 19	OD: Healed corneal ulcer with minimal scarring, OS: No reaction in the anterior chamber		OD: topical antifungal discontinued, OS: ED moxifloxacin + dexamethasone QID, ED timolol + dorzolamide BID.

## Discussion

The incidence of fungal corneal ulcers caused by many fungi previously considered non-pathogenic for humans has markedly increased due to the use of topical corticosteroids [7,8]. *Aureobasidium *is also a saprophyte present in the environment and rarely causes opportunistic infection in humans [9].

*Aureobasidium pullulans* is a yeast-like fungus with melanin in its cell wall [9,10]. The *Aureobasidium *genus belongs to the phylum Ascomycota, the order Dothideales, and the family Dothideaceae [9,10]. It is a ubiquitously distributed saprophyte, particularly found in humid environments, and commonly isolated from plant debris, soil, wood, and fabrics [9]. It is a common contaminant in clinical laboratories that frequently colonises on moist stone and glass [8]. It occasionally causes phaeohyphomycosis in humans [9,10].

*Aureobasidium pullulans* is the most commonly reported species associated with opportunistic diseases in humans; other clinically significant species include *Aureobasidium proteae*, *Aureobasidium melanigenum*, and *Aureobasidium mansoni* [9-14]. The infection is common among immunocompromised and diabetic patients [15]. It has been reported to cause localised cutaneous infections, corneal and scleral infections, and abscesses in the spleen and jaw to peritonitis, pneumonia, meningitis, and septicemia (fungemia) [6,9,15]. This fungus commonly occurs in hospital environments and has an affinity for synthetic materials and surgically implanted devices [9,15]. Pritchard and Muir, in a five-year review of 556 dematiaceous hyphomycetes, found seven pathogenic strains of *Aureobasidium pullulans*, all of which were associated with fungal peritonitis in patients undergoing chronic ambulatory peritoneal dialysis [16]. A recurrence of *Aureobasidium pullulan* peritonitis was reported after medical therapy alone, and complete eradication of infection was achieved after removal of the peritoneal dialysis catheter. Fungal colonies were seen as black dots on the inner walls of the catheter [15,17].

The clinical diagnosis of *Aureobasidium *infection is usually not suspected until the fungus is isolated from clinical specimens [9]. Several times, it is identified after the death of patients when postmortem samples are investigated [9]. Microbiological identification of the organism is also difficult. Direct microscopy of clinical specimens is not described for the identification of this organism [9]. *Aureobasidium *can be isolated using culture media such as malt extract agar, cornmeal agar, Sabouraud dextrose agar, glucose peptone agar, etc. *Aureobasidium *grows rapidly, producing flat and smooth growth, which soon becomes covered with slimy exudates at 30°C incubation [9]. Initially, the colonies are cream, yellow, or pink in colour, later becoming green, brown, and black [9, 17]. The microscopical appearance is vegetative hyphae (3-12μm wide), septate, hyaline, locally converting into blackish-brown, thick-walled chlamydospores, and expanding hyphae with irregular dichotomous branching [9]. The conidia-bearing cells appear undifferentiated and mostly intercalary in hyaline hyphae [9]. Histopathological similarities are observed with other dematiaceous fungi that cause phaeohyphomycosis, such as *Hormonema dematiodes* [9]. *Aureobasidium *produces synchronous blastoconidia only from hyaline hyphae, which differentiates it from *Hormonema dematiodes* [9]. *Aureobasidium pullulans* can be differentiated from other dematiaceous fungi by genotyping: deoxyribonucleic acid (DNA) sequencing or restriction fragment length polymorphism (RFLP) analysis [9].

The first documented case of keratomycosis caused by *Aureobasidium pullulans* was reported by Ashikaga in 1920 in a 29-year-old logger in Japan [7]. In 1974, Jones and Christensen reported the second case of *Aureobasidium pullulans *causing a corneal ulcer and the organism’s sensitivity to antifungal drugs [7]. The patient was struck in the OD with a piece of dirt from a shovel two weeks ago [7]. He was treated with an antibiotic-corticosteroid ointment for five days before referral to an ophthalmologist [7]. However, both amphotericin and natamycin showed similar susceptibility on the basis of in vitro tests; no clinical improvement was noted with amphotericin B [7]. This could be due to the viscosity and adherence of natamycin to the ulcer surface, as noted in the treatment of Fusarium ulcers [7]. We found only two additional cases of *Aureobasidium pullulans* keratitis reported in the literature, both following corneal procedures [14]. Maverick reported a case of *Aureobasidium pullulans* keratitis following refractive laser epithelial keratomileusis, which was unresponsive to conventional therapies. The patient was treated with topical natamycin and systemic itraconazole [6]. In the present case, the patient also approached us for treatment of a corneal ulcer not responding to conventional therapy. However, there was no history of ocular trauma or surgery. The source of infection is unclear in this case; it seems likely to be an unnoticed, trivial injury, as he used to visit the countryside home with domestic pets, although he does not work in fields. In addition, there was no associated ocular or systemic condition predisposing to opportunistic infection. The patient had a two-month-long course of topical antifungal and antibiotic therapy without any significant improvement. However, microbiological investigations were not recommended. We performed corneal scraping for smears and culture, which revealed growth of *Aureobasidium pullulans* after three weeks of incubation. A good response was achieved with topical natamycin and systemic itraconazole. In lower-middle-income countries like India, primary and secondary care providers frequently start broad-spectrum antibiotics and antifungal therapy for infectious keratitis without bacterial and fungal cultures. A favourable outcome is achieved if the causative organism is susceptible to the given treatment; however, this is not always the case, as it can result in worsening or even perforation of the cornea. 

Ideally, microbiological investigations should be recommended before the commencement of antifungal/antibacterial therapy in patients with infectious keratitis. However, it takes a long time, especially in fungal culture; it may be three or four weeks, and direct microscopic detection of fungi in corneal scrapings stained by various methods is not always possible because of low sensitivity and easy misinterpretation and common artefacts [5]. Therefore, a rapid, presumptive diagnosis based on typical clinical features is important [5].

The clinical diagnosis of *Aureobasidium *keratitis or other dematiaceous keratitis is difficult, as clinical manifestations of fungal keratitis are similar regardless of the causative agent, leading to a delay in diagnosis. However, the severity and progression of the infection may vary [1, 5]. Fusarium keratitis is usually severe, tends to progress rapidly, and may lead to deep lesions and perforation. *Aspergillus *keratitis is moderately severe, does not progress so rapidly, and is more responsive to therapy than *Fusarium *keratitis. Dematiaceous keratitis is usually a low-grade, smouldering keratitis lasting for several weeks with minimal inflammation or structural alterations as seen in keratitis due to *Curvularia *or *Phialophora *species [5]. However, *Botryodiplodia* (*Lasiodiplodia*) *theobromae *can cause more severe infection, comparable to *Fusarium *keratitis [1]. Keratitis caused by* Candida albicans* presents as a small, sharply demarcated area of epithelial ulceration, a discrete, expanding stromal infiltration, an endothelial plaque, and a hypopyon seen early in the course [5].

Recent in vitro studies have shown that these organisms are susceptible to amphotericin B, posaconazole, and itraconazole; resistant to fluconazole (>64 µg/ml); and have high minimum inhibitory concentrations (MICs) of voriconazole, isavuconazole, caspofungin, and micafungin [10]. Fluconazole showed variable susceptibility against *Aureobasidium pullulans*, with both low (4 µg/ml) and high (64 µg/ml) MICs reported in two case studies [10,15].

The standard antifungal regimen for the treatment of *Aureobasidium *infection is not defined, as very few clinical case reports and case series are documented in the literature. The available data from previous reports suggested both single and combination-therapy approaches. Amphotericin B alone is used effectively for the treatment of meningitis and peritonitis with catheter removal [13,17]. Voriconazole and fluconazole alone were used to treat disseminated nosocomial fungal infection by *Aureobasidium pullulans* [9,18]. A successful outcome was reported with amphotericin B switched to itraconazole due to toxicity in a patient with chronic lymphocytic leukaemia [19]. A combination of fluconazole and amphotericin B has been used successfully in the treatment of *Aureobasidium* pneumonia and septicaemia [20].

*Aureobasidium pullulans *keratitis responds well to treatment with topical and systemic antifungal therapy. It is successfully treated with topical natamycin or fluconazole alone or with systemic itraconazole and fluconazole [6,7]. Repeated surgical debridement is useful for better drug penetration with topical therapy. Before the commencement of antifungal/antibacterial therapy, the severity of the keratitis should be graded using existing grading systems, taking into account lesion progression, the diameter and depth of the ulcer, suppuration, the presence of hypopyon, and perforation [5]. Topical itraconazole has been reported ineffective in severe fungal keratitis, possibly due to its poor water-solubility and consequent poor corneal stromal penetration [5]. In this patient, significant improvement was not observed despite the use of three topical antifungal agents (natamycin, amphotericin B, and itraconazole). Systemic antifungal agents are useful in severe fungal keratitis [5].

The clinical correlation between fungal keratitis in the OD and the sudden inflammation in the OS is not clear in this patient. The patient was immunocompetent, and the left cornea was intact. At the time when the OD had a corneal ulcer, the patient underwent an OS peripheral iridotomy, which may have induced the iritis that precipitated later on, or the patient may have had an unnoticed pre-existing chronic anterior uveitis. The patient was also instilling a combination eye drop of brinzolamide and brimonidine in both eyes. Brimonidine can also induce inflammation. However, it was discontinued when he presented here. A detailed clinical evaluation and its proper documentation in the prescriptions are very important for the accurate diagnosis and appropriate management of any disease, which were unfortunately not available in this case.

## Conclusions

*Aureobasidium pullulans* keratitis is a rare fungal keratitis that usually occurs following ocular trauma or surgery. However, it was not preceded by ocular trauma or surgery in this patient. *Aureobasidium pullulans* keratitis may be suspected in cases of corneal ulcers that do not respond to conventional therapy. For optimal treatment outcome, an early provisional diagnosis should be made based on typical clinical features. A careful microbiological examination is important to identify the causative organism. *Aureobasidium pullulans* keratitis can be effectively treated with topical natamycin and systemic itraconazole, as reported in this case. Further case studies or case series are needed to establish standard management.
